# miRNAs reshape immunity and inflammatory responses in bacterial infection

**DOI:** 10.1038/s41392-018-0006-9

**Published:** 2018-05-25

**Authors:** Xikun Zhou, Xuefeng Li, Min Wu

**Affiliations:** Department of Biomedical Sciences, University of North Dakota, Grand Forks, ND 58202-9037 USA

## Abstract

Pathogenic bacteria cause various infections worldwide, especially in immunocompromised and other susceptible individuals, and are also associated with high infant mortality rates in developing countries. MicroRNAs (miRNAs), small non-coding RNAs with evolutionarily conserved sequences, are expressed in various tissues and cells that play key part in various physiological and pathologic processes. Increasing evidence implies roles for miRNAs in bacterial infectious diseases by modulating inflammatory responses, cell penetration, tissue remodeling, and innate and adaptive immunity. This review highlights some recent intriguing findings, ranging from the correlation between aberrant expression of miRNAs with bacterial infection progression to their profound impact on host immune responses. Harnessing of dysregulated miRNAs in bacterial infection may be an approach to improving the diagnosis, prevention and therapy of infectious diseases.

## Microbiology: Tracking infection with tiny transcripts

Changes in production of tiny cellular RNAs in response to bacterial infection could guide the development of better diagnostics and therapies. MicroRNAs regulate other genes by binding to messenger RNA strands and controlling their translation into proteins. Xikun Zhou, Min Wu and colleagues of the University of North Dakota have now reviewed current knowledge about how microRNA levels shift during infection with various bacterial pathogens. These microRNAs can modulate the immune response as well as pathways that influence metabolic activity and cell survival. Increasing studies have indicated that shifts in microRNA levels in response to different infections could provide a potential bacterial ‘fingerprint’ for achieving accurate diagnosis. With deeper insight into how different microRNAs influence infection, it might one day day become possible to target these molecules with ‘antisense’ or ‘agonist’ drugs that modulate their activity.

## Introduction

Pathogenic bacteria hold a wide range of strategies to invade, survive, and replicate in their hosts. These pathogens are the major causes of many deadly diseases and widespread epidemics in mammals, including humans. However, host immune systems have also developed extremely complex adaptations to counteract bacterial infection^1^. Host–pathogen interactions are one of the most complex themes involved in disease initiation, development and progression. An intact immune system is critical for host resistance to bacterial infections. There are many important regulators involved in a range of pathological processes during host defense against infection that modulate diverse biological processes. Host immune cells, such as lymphocytes, innate lymphoid cells, macrophages and neutrophils, are vital parts of innate immunity systems that find, process and clear invading microbes by phagocytosis, secreting cytokines and mounting inflammatory responses. Pathogen-associated molecular patterns (PAMPs) bind and talk with Toll-like receptors (TLRs), NOD-like receptors (NLRs) and other pattern-recognition receptors (PRRs) to activate a number of inflammatory signals and subsequently lead to proinflammatory cytokine production or inflammatory cell death. Each type of PAMP can be recognized by its specific receptor(s)^2,3^. Subsequently, the adaptive immune response is induced to promote and facilitate the removal of pathogenic bacteria^4^. Once these intruders are cleared, negative immunoregulatory cytokines and Th2 cells play a dominant role in balancing the extent of the immune response to avoid overreaction and tissue damage^5^. Recent studies provided some insight into the critical participation of microRNAs (miRNAs) in host immune defense against bacterial infection.

miRNAs are evolutionarily conserved small (~22 nucleotide) non-coding RNAs first discovered two decades ago^6^. The transcription of miRNAs is most commonly mediated by RNA polymerase II; they are then processed by two nucleases, Drosha and Dicer. After exporting to the cytoplasm, the functional mature miRNA is incorporated into and preferentially stabilized by the RNA-induced silencing complex (RISC). In most cases, the RISC converts the miRNA to a 6–8 nucleotide-long complementary region, named the ‘seed sequence’, on the 3′-Untranslated Region (3′-UTR) of its target mRNA and mediates its function. The partial or imperfect complementarity of a miRNA to a target mRNA may result in translational repression, while full or perfect complementarity binding sites cause target degradation at the posttranscriptional levels^7^. Certain miRNAs can also bind the 5′ untranslated region (5′-UTR) and amino-acid coding sequence (CDS) sites of their target mRNA, and several miRNAs may also induce gene expression^8–10^. Moreover, one mRNA might be modulated by numerous miRNAs, and a miRNA has the ability to modulate the expression of a number of target mRNAs. miRNAs have emerged as critical regulators in a great deal of biological processes, such as cell proliferation, differentiation, autophagy, metabolism and immune responses. The dysregulated expression of miRNAs has also been correlated with various diseases, including cancer, autoimmunity, and cardiovascular diseases, among others ^11^.

In this review, we first summarize the dysregulated miRNAs identified during different bacterial infections. Then, we describe the host signal transduction pathways utilized by bacterial effectors by which miRNA expression is dysregulated in mechanisms of modulation. Finally, we discuss the potential of miRNAs to serve as diagnosis biomarkers and treatment targets, and discuss the challenges facing miRNA studies.

## MiRNAs affected by bacterial infections

Bacterial pathogens are thought to have complex interactions with relevant hosts, and the interactions between hosts and pathogens are becoming a forefront research area of infectious diseases. Latest studies have highlighted that the expression of miRNAs is profoundly impacted by a variety of bacterial pathogens and that likewise miRNAs impose strong pressure to the invading microorganisms.

### *Helicobacter pylori*

*H. pylori* is particularly capable of colonization in human stomach and is thus responsible for various gastric diseases, such as chronic active gastritis, peptic ulcers, and gastric carcinoma worldwide^12,13^. Several studies have reported that infection of gastric epithelial cells with *H. pylori* could lead to altered expression of miRNAs, including let-7^14–16^, miR-30b^17^, miR-210^18^, miR-1289^19^, miR-152/miR-200b^20^, miR-155^21–25^, miR-16, and miR-146a^24–26^. Histological analysis has shown higher miR-155 levels in gastric mucosal tissue sections of patients infected with *H. pylori*. Potential binding sites for nuclear factor-κB (NF-κB) as well as activator protein-1 (AP-1) have been identified within the BIC/miR-155 promoter, and both NF-κB and AP-1 are the necessity for the induction of miR-155 upon *H. pylori* infection in gastric epithelial cells^24^. The expression of miR-155 might also be influenced by Foxp3 in *H. pylori*-infected T cells^23^. In addition to NF-κB signaling in bone marrow-derived macrophages (BMDMs), TLR2/4- and NOD1/2-independent upregulation of miR-155 was found to depend on the *H. pylori* type IV secretion system (T4SS)^21^. Several miR-155-targeted mRNAs, including tumor protein p53-inducible nuclear protein 1 (TP53INP1), tetraspanin 14 (Tspan14), lipin 1 (Lpin1), phorbol-12-myristate-13-acetate-induced protein 1 (Pmaip1), protein kinase (cAMP-dependent, catalytic) inhibitor alpha (PKIα), IκB kinase ε (IKK-ε), Sma- and Mad-related protein 2 (SMAD2), and Fas-associated death domain protein (FADD), have been linked to proapoptotic and immune responses^21,23,24,27^. miR-155 knockout mice failed to control *H. pylori* infection and had reduced protection from infection after *H. pylori*-specific vaccination than their wild-type counterparts as a result of impaired pathogen-specific T helper type 1 (Th1) and Th17 responses^22^. A multi-epitope vaccine, CTB-UE, could relieve the *H. pylori*-induced gastric inflammatory reaction by upregulating miR-155 to inhibit Th17 responses^28^. These results implied that the increase in miR-155 expression during *H. pylori* infection is involved in negative regulation of inflammation by attenuating NF-κB signaling, Th17/Th1 differentiation and cyclic adenosine monophosphate (cAMP) activity^23,24^. Furthermore, there was a strong link between miR-155 levels and immunohistochemical grades in *H. pylori*-positive patients, and miR-155 expression was downregulated in intestinal metaplasia individuals^29^.

Another miRNA, miR-146a, has been shown to be increased after *H. pylori* infection in gastric epithelial cells, as well as in gastric mucosal tissues, in an NF-κB-dependent manner. Subsequently, miR-146a can diminish the expression of target genes, e.g., TNF receptor-associated factor 6 (TRAF6) and IL1 receptor-associated kinase 1 (IRAK1). In addition, miR-146a may inhibit the expression of IL-8, growth-related oncogene (GRO)-α, and macrophage inflammatory protein (MIP)-3α, TNF-α, and IL1β by reducing NF-κB activity^30,31^. Additional studies have found that the overexpression of miR-146a results in significantly reduced Prostaglandin endoperoxide synthase 2 (PTGS2) production induced by *H. pylori* infection^26^.

*H. pylori* infection is thought to have a link with the early stages of gastric cancer pathogenesis via the induction of chronic gastritis^16^. This is also a hallmark of *H. pylori*, making it different from other bacteria. Cytotoxin-associated gene A (*CagA*), a key virulence factor of *H. pylori*, harms the gastric mucosa and is associated with an increased risk of atrophic gastritis, peptic ulcer and gastric cancer^13,16^. Hayashi et al. found that CagA drives epigenetic regulation to impede let-7 expression in *H. pylori*-related carcinogenesis. CagA augmented c-myc, DNA methyltransferase 3B (DNMT3B) and Enhancer of Zeste homolog 2 (EZH2) expression and reduced miR-26a and miR-101 levels, lowering let-7 expression by altering histone and DNA methylation^16^. CagA can also downregulate miR-370 levels, resulting in increased FoxM1, a positive modulator for cell growth^32^. miR-320a and miR-4496 attenuate the possibility of CagA-induced cancer-initiation and chemoresistance through influencing β-catenin and ATP-binding cassette subfamily G member 2^33^. miR-210 was downregulated in the gastric epithelium in response to persistent *H. pylori* infection. Inflammation-induced epigenetic silencing of miR-210 augmented cell proliferation by activating two tumorigenesis-related proteins, STMN1 (stathmin1) and DIMT1 (demethyladenosine transferase 1)^18^. Several other miRNAs, such as miR-203^34^, miR-204^35^, miR-375^36^, and miR-27b^37^, were aberrantly expressed in *H. pylori-*positive tissues and cells and affecting neoplastic transformation and invasion. These studies suggest a role for miRNAs in regulating pathogenesis in various *H. pylori*-infected cell types.

### *Salmonella*

*Salmonella* is a Gram-negative intracellular pathogen belonging to the family Enterobacteriaceae and can cause a number of diseases in humans and animals, such as gastroenteritis and typhoid fever^38^. *Salmonella* has three main serovars, *typhi*, *typhimurium*, and *enteritidis*, and can exert diverse effects to establish an intracellular niche for successful propagation^39^. A number of studies have reported that the dysregulation of miRNAs critically contributes to disease pathogenesis. Hoeke et al. showed that focal adhesion and organization of the actin cytoskeleton is regulated by miRNAs in an intestinal *Salmonella typhimurium* infection model. *S. typhimurium* infection upregulated the expression of miR-29a, which subsequently targeted Caveolin 2, a focal adhesion factor that is associated with uptake of bacterial pathogens, to modulate the activation state of the small Rho GTPase CDC42^40^. miR-146 decreased the induction of six members of the apolipoprotein gene family in *S. typhimurium*-infected zebrafish embryos. This suggested that miR-146 may play a role in regulating lipid metabolism during inflammation^41^. TLR4-sensing of bacterial LPS downregulated the expression of let-7 family miRNAs upon Salmonella infection. The downregulation of these miRNAs promoted the expression of the key cytokines IL6 and IL-10^42^. By a combination of high-throughput screening with a library of miRNA mimics and RNA-seq, Maudet et al. proposed that miRNAs are potential modulators in *S. typhimurium* infection and that distinct miRNAs impair various infection stages. They further found that downregulation of the miR-15 family upregulated cyclin D1 expression upon Salmonella infection. G2/M arrest of host cells dramatically increased Salmonella replication within hosts^43^. Moreover, miR-155 regulated the function of both lymphocytes and DCs, leading to an overall diminution of immune responses. Vaccination of miR-155-deficient mice with an attenuated vaccine against *S. typhimurium* failed to protect them against virulent *S. Typhimurium* ^44^.

Macrophage colony-stimulating factor (M-CSF, CSF1) is a cytokine for attracting macrophages to infection sites to defend against different pathogenic infections. Virulent *Salmonella enteritidis* modulates intestinal epithelial miR-128 levels, which inhibits epithelia-secreted M-CSF and impedes the subsequent recruitment of macrophage^45^. A combined study of differentially expressed miRNAs with mRNAs predicted miRNA targets, revealing miRNA-mRNA profiles. This analysis found that miR-214 and miR-331-3p could participate in host immunity against *S. typhimurium*. Salmonella-challenged pigs showed downregulated miR-214 expression and upregulated miR-331-3p expression in whole blood. While levels of the candidate targets (SLC11A1 and PIGE-108A11.3) of miR-214 were enhanced following challenge, the potential target (VAV2) of miR-331-3p was reduced^46^. Another binding site enrichment analysis of miRNAs responsible for deregulated mRNAs in peripheral blood suggested that miR-143 and miR-26 might be involved in the initiation and progression of Salmonella infection in pigs^47^. In addition, miRNAs may also modulate the innate immunity involving miRNAs to *S. enteritidis* infection in laying chicken ceca^48^. Thus, these deregulated miRNAs may be functionally important for manipulating Salmonella-induced inflammation.

### *Pseudomonas aeruginosa*

*Pseudomonas aeruginosa* is an important opportunistic Gram-negative bacterium that infects a broad range of individuals, invading many different parts of the body, with corresponding symptoms and signs^49^. Multidrug-resistant Pseudomonas can be deadly for patients in intensive care units worldwide^50^. Up to date, few miRNAs have been reported in modulating inflammatory responses, and are likely TLR/NF-κB-responsive^51^. Our laboratory recently reported that miR-302b can be activated by TLR2 and TLR4 via ERK-p38-NF-κB pathways following *P. aeruginosa* infection. miR-302b, together with other members of the miR-302 family, is a crucial regulator of TLR-induced downstream NF-κB signaling, macrophage and epithelial cell activation, and respiratory inflammation via directly targeting of IRAK1, a member of the TLR/myeloid differentiation factor 88 (MyD88) complex that is critical for NF-κB activation^52^. We further identified another miRNA, miR-301b, can be induced via a TLR4/MyD88/NF-κB pathway against *P. aeruginosa* infection. miR-301b positively modulates the expression of the anti-inflammatory cytokines IL-4 and TGF-β1 and negatively regulates the expression of the proinflammatory cytokines MIP-1α and IL-17A. This function is exerted by repressing c-Myb expression, and the antimicrobial effect was potentiated by caffeine uptake. Moreover, repression of miR-301b resulted in elevated levels of neutrophil infiltration ^53^.

There are several other negative feedback miRNAs, such as miR-762 and miR-155, whose levels are enhanced upon *P. aeruginosa* infection to downregulate levels of immune response genes^51,54^. Using a *P. aeruginosa*-infected *Caenorhabditis elegans* model, Ren et al. reported that the let-7 family (let-7-Fam) acted in innate immune response pathways to timely induce strong immunity to reduce pathogen-induced stress. The developmental timing phenotypes of let-7-Fam miRNA mutants were modified by growth in pathogenic processes. The let-7-Fam miRNA activity was downmodulated during *P. aeruginosa* infection through the p38 MAPK signals. Furthermore, let-7-Fam miRNAs-reduced resistance to pathogens, also involving the p38 MAPK axis^55^. In *C. elegans*, let-7 may influence innate immunity against *P. aeruginosa* PA14 infection in both the intestine and the neurons^56^. Muraleedharan et al. found that the miR-183/96/182 cluster modulated the immune response in cornea to bacterial infection via influencing the neuroimmune axis. Expression of miR-183/96/182 in macrophages decreased, while reducing or blocking miR-183/96/182 in macrophages and polymorphonuclear neutrophils (PMNs) increased their ability to phagocytize and kill *P. aeruginosa*^57^.

One important feature of *P. aeruginosa* is its much higher frequency of infections in patients with cystic fibrosis (CF) than most other patient groups and healthy individuals^58^. miRNA profiles for CF bronchial epithelial IB3-1 cells after *P. aeruginosa* challenge demonstrated that miR-93, which is highly expressed in basal conditions, reduced along with increased IL-8 levels after infection. Specifically, in addition to increased IL-8 transcription upon NF-κB activation, IL-8 protein levels were modulated via IL-8 mRNA crosstalk with miR-93 at posttranscriptional levels^59^. The unfolded protein response (UPR) has been reported to play an important role in innate immunity and inflammation, involving the development, differentiation, and survival of immune cells^60^. *P. aeruginosa* infection upregulated the levels of miR-233 via p38 MAPK circuits. miR-233 reportedly also has an impact on innate immune response through activation of a UPR-associated protein, sarco/endoplasmic reticulum Ca^2+^-ATPase (SERCA)^61^. Future studies may elucidate whether miRNAs have therapeutic efficacies in the treatment of CF.

### *Mycobacterium*

Tuberculosis (TB) is a common infectious disease, with morbidity and mortality rates exceeding tens of millions of people each year. Both the intracellular bacteria *Mycobacterium tuberculosis* (Mtb) and *Mycobacterium bovis* (*M. bovis*) can infect animals and humans and are the most well-investigated mycobacteria^62^. Concerning innate immune responses, Dorhoi et al. demonstrated that miR-223 controlled tuberculosis susceptibility via impacting recruitment of neutrophils through chemokine (C-C motif) ligand 3 (CCL3), chemoattractant chemokine (C-X-C motif) ligand 2 (CXCL2), and IL6 in myeloid cells. Deletion of miR-223 increased susceptibility to lung infection of Mtb-resistant mice^63^. Mtb could induce the expression of miR-99 in dendritic cells (DCs). Importantly, Inhibiting miR-99b in DCs dramatically augmented levels of proinflammatory cytokines including IL1β, IL-12, and IL6, and decreased the bacterial burden^64^. Regarding adaptive immune responses, infection of mice with *Listeria monocytogenes* or vaccine strain *M. bovis* bacillus Calmette-Guerin (BCG) largely reduced expression levels of miR-29 in CD8^+^ T cells, CD4^+^ T cells, and natural killer cells. miR-29 ihibited IFN-γ production via targeting of IFN-γ mRNA. Thus, miR-29 transgenic mice exhibited stronger Th1 responses and higher resistance to BCG or Mtb infection ^65^.

Hedgehog (HH) signaling is an important factor for cell fate decisions in various disease conditions. *M. bovis* BCG-specific TLR2 signaling influences the states of Sonic HH (SHH) signaling in macrophages through TNF-α secretion. Intriguingly, SHH signaling serves as a negative regulator to counteract TLR2 responses in mycobacterial invasion. SHH signaling drives miR-31 and miR-150 expression, which modulates TLR2 levels involving MyD88, a canonical adapter for TLR signaling ^66^.

As an important miRNA for immune reactions, miR-155 is also increased in macrophages after Mtb and *M. bovis* BCG infection. Enhancing miR-155 expression augments autophagic influx in macrophages, thereby facilitating mycobacterial phagosome maturation and ROS production and decreasing the survival rate of intracellular mycobacteria^67,68^. Mice with miR-155-deficiency died much earlier and showed drastically increased colony forming units (CFUs) in their lungs than wild-type mice after Mtb infection^69^. However, miR-155 could also facilitate the survival of Mtb in macrophages by directly attenuating the expression of BTB and CNC homology 1 (Bach1), a transcriptional repressor of haemoxygenase-1 (HO-1), and SH2-containing inositol 5′-phosphatase 1 (SHIP1), which is important for Mtb survival^70^. Rothchild et al. dissected the in vitro and in vivo function of miR-155 in impacting both innate and adaptive immunity systems. miR-155 helped improving survival of Mtb-infected macrophages but providing a niche supporting bacteria colonization. However, the miRNA also extended the survival and augmented the function of Mtb-specific T cells to upregulate adaptive immunity. Although miR-155 may render early defense, miR-155-deficient mice may succumb in the late stages of infection ^71^.

The dynamic expression and function of miRNAs and their isoforms in infection are an important focus in the field of host-Mtb interactions. Siddle et al. performed a genome-wide miRNA transcriptional analysis of human DCs exposed to mycobacteria and other bacteria with different virulence. They revealed some critical elements of miRNA variants in immune reaction against bacterial infection, particularly identifying miR-132/212 family as a vital responder to mycobacteria. Another insight is that infection might differentially impact the expression of each member of the same polycistronic family ^72^.

M1 and M2 macrophages are functionally polarized subsets of macrophages in various conditions including bacterial infection. Kruppel-like factor 4 (KLF4) is a key molecule for regulating this polarization^73^. During Mtb infection, downregulation of miR-26a upregulated KLF4, which in turn prevented trafficking of Mtb to lysosomes^74^. Additionally, infection of macrophages with Mtb and *M. bovis* BCG led to higher expression of miR-125a^75^, miR-132^76^, miR-26a^76^, miR-146a^77,78^, and miR-21^79^, which functioned as negative regulators, whereas miR-206^80^ as a positive regulator, decreased the expression of miR-let-7f^81^ in host defense. A nuclear body protein, Sp110 that has been linked to TB resistance, could modulate expression levels of miRNA in macrophages, hence modifying host immune response (miR-146a, miR-155, miR-27b and miR-29a) and apoptosis (miR-125a) in response to infection with Mtb^82^. Therefore, miRNAs may be emerging mediators of macrophage inflammatory responses to bacterial pathogens.

### *Listeria* monocytogenes

*Listeria monocytogenes* is an intracellular bacterium that causes serious illness in immunocompromised individuals and pregnant women^83^. *L. monocytogenes* can evade miRNA-mediated host defense in various cells. miR-146b, miR-16, let-7a1, miR-145, and miR-155 were significantly dysregulated following Listeria infection in epithelial cells^84^. Schnitger et al. demonstrated that *L. monocytogenes* promoted significant changes in miRNA expression in macrophages. miR-146a, miR-155, miR-125a-3p/5p, and miR-149 were among the most altered miRNAs. miR-125a-3p/5p were found to be involved in the TLR2 axis, while transactivation of miR-155 upon infection was influenced by NF-κB p65^83^. Lind et al. further showed that CD8^+^ T cells with miR-155 deficiency exhibited unresponsiveness to the AKT signaling after T-cell receptor (TCR) cross-linking responses to *L. monocytogenes* infection. This suggested that miR-155 is necessary for agitating a proper CD8^+^ T-cell response^85^. Strikingly, miR-29 inhibited immune responses of natural killer cells, CD4^+^ T cells and CD8^+^ T cells to *L. monocytogenes* infection by targeting IFN-γ^65^. In contrast, in macrophages, miR-21 limited the uptake of listeria monocytogenes to control infection by impairing the intracellular niche.

The intestinal tract is thought to be the main reservoir of microbes in humans, but much remains to be determined about the role of the intestinal microbiota in modulating miRNA expression. Archambaud et al. showed that intestinal microbiota could alter the gut miR-143, miR-148a, miR-200b, miR-200c, and miR-378 responses after oral Listeria infection. Moreover, the expression levels of protein-coding target genes were inversely correlated with those of the above-mentioned miRNAs^86^. Thus, miRNAs may mediate the proinflammatory responses of host immune responses to *L. monocytogenes* infection.

### *Staphylococcus aureus*

*Staphylococcus aureus*, a Gram-positive round-shaped bacterium, causes common skin infections, and occasionally causes pneumonia, endocarditis, and osteomyelitis, in humans^87^. *S. aureus* produces a spectrum of virulence factors and modifies the protein levels of TGF-β, which may limit the inflammation and tissue injury during infection^88^. TGF-β induces expression of miR-29b to promote murine alveolar macrophage dysfunction, and miR-29b compromises bacterial killing in macrophages through prostaglandin E2 (PGE2) signaling^89^. A network of miRNA-gene-pathway interactions could be induced in bovine mammary gland cells in response to invading *S. aureus*. The increase in bta-miR-223 and bta-miR-21-3p was found in the teat quarters following high dose *S. aureus* infection. Further analysis suggested critical roles of these two miRNAs in defending hosts against bacterial infection, probably through inhibiting CXCL14 and KIT proto-oncogene receptor tyrosine kinase (KIT)^90^. Skin wound healing after *S. aureus* infection might be impaired in miR-142-deficient mice compared to that in wild-type mice. These alterations may be associated with cytoskeletal function, and the levels of the small GTPases were thus drastically enhanced in miR-142-deficient neutrophils^91^. Bacterial pneumonia after influenza infection is dubbed with high mortality and morbidity^92,93^. In contrast to the results from other murine infection models^44,85^, mice with miR-155 deficiency were resistant to infection, with significantly reduced bacterial CFUs and no differences in viral load, along with augmented IL-23 and IL-17 compared to WT mice after sequentially challenged by virus and bacteria, respectively. A miR-155 antagomir application significantly reduced bacterial loads versus control antagomir treatment after sequentially infected by viruses and bacteria^92^. These studies indicated that the regulation of miR-155 may be modified under different infection conditions.

### Other bacterial pathogens

In addition to the above-mentioned infection models, miRNAs have been reported to be involved in host immune responses against the other bacterial pathogens. Despite NF-κB activation being the necessity of miR-155 induction, much remains to be elucidated about the underlying mechanisms of miR-155 induction by various stimuli or pathogens. Cremer et al. showed that de novo synthesis of c-Jun and c-Fos upon NF-κB activation is needed for inducing miR-155 by *Francisella novicida*, *Burkholderia cenocepacia*, and *Mycobacterium smegmatis* stimuli in monocytes^94^. miR-155 is also critical for effective clearance of primary and secondary *Streptococcus pneumoniae* colonization via IL-17A and IFN-γ CD4^+^ T-cell responses^95^. Interestingly, miR-155 may not participate in the cytokine production induced following *Francisella tularensis* phagocytosis. Instead, this miRNA may be important for inhibiting endotoxin-stimulated TNF-α secretion^96^. In *P. gingivalis*-infected BMMs, mmu-miR-155-5p could markedly decrease the production of TNF-α^97^. Thus, these data demonstrate that miR-155 may act as a global negative regulator of inflammation during bacterial infection.

miR-15a and miR-16 have been reported to play an important role in bacterial infection-associated sepsis. Deletion of miR-15a/16 in myeloid cells significantly decreased *E. coli*-associated mortality in several mouse models of sepsis. Consistently, miR-15a/16 overexpression using miRNA mimics decreased both phagocytosis and production of mitochondrial reactive oxygen species. In addition, deficiency of miR-15a/16 boosted secretion of cytokine/chemokine of bone marrow-derived macrophages (BMDMs) at the initial phase of infections^98^. This is different from miR-15a/16, presumably a restriction factors for *Salmonella* infection via control of the G1/S phase transition^43^.

In *Neisseria gonorrhoeae* infection models, TLR4 instead of TLR3 is required for inducing miR-718 expression in macrophages. miR-718 can impact PI3K/AKT axis through direct downregulation of phosphatase and tensin homolog (PTEN), while increasing AKT phosphorylation and cytokine production. Decrease in miR-718 levels correlated to bacterial burdens during *N. gonorrhoeae* infection and thus altering the infection dynamics of *N. gonorrhoeae* in vitro^99^. Additionally, miR-214 could be significantly upregulated by *Vibrio harveyi* and LPS stimulation. Upregulating miR-214 subsequently inhibited the production of inflammatory cytokines by targeting MyD88 to avoid excessive inflammation^100^. However, *Chlamydia muridarum* infection in mouse genital tracts drastically reduced miR-214, while repressing the expression of intracellular adhesion molecule 1 (ICAM1). The alteration of ICAM1 by miR-214 in mice correlated with a reduction of neutrophil infiltration in genital tissues^101^. Filopodia are thin actin-rich cell protrusions, and forming host-cell filopodia is critical for phagocytosis and bacterial internalization. miR-29b-2-5p, by inhibiting its direct target UNC5C, rapidly increased filopodia in hosts upon *Shigella flexneri* infection^102^. These studies highlight the complicated transcriptional and posttranscriptional response mechanisms of host cells to bacterial infection.

## Significance of altered miRNAs in bacterial infection

Host response to pathogens needs fine regulation of various cellular signals including immune signaling^103^. Given the abnormal miRNA expression in bacterial infection, it has been hypothesized that these dysregulated miRNAs could affect multiple cell physiological functions and pathological processes, depending on their target genes.

### TLRs/NF-κB signaling

**Fig. 1:**
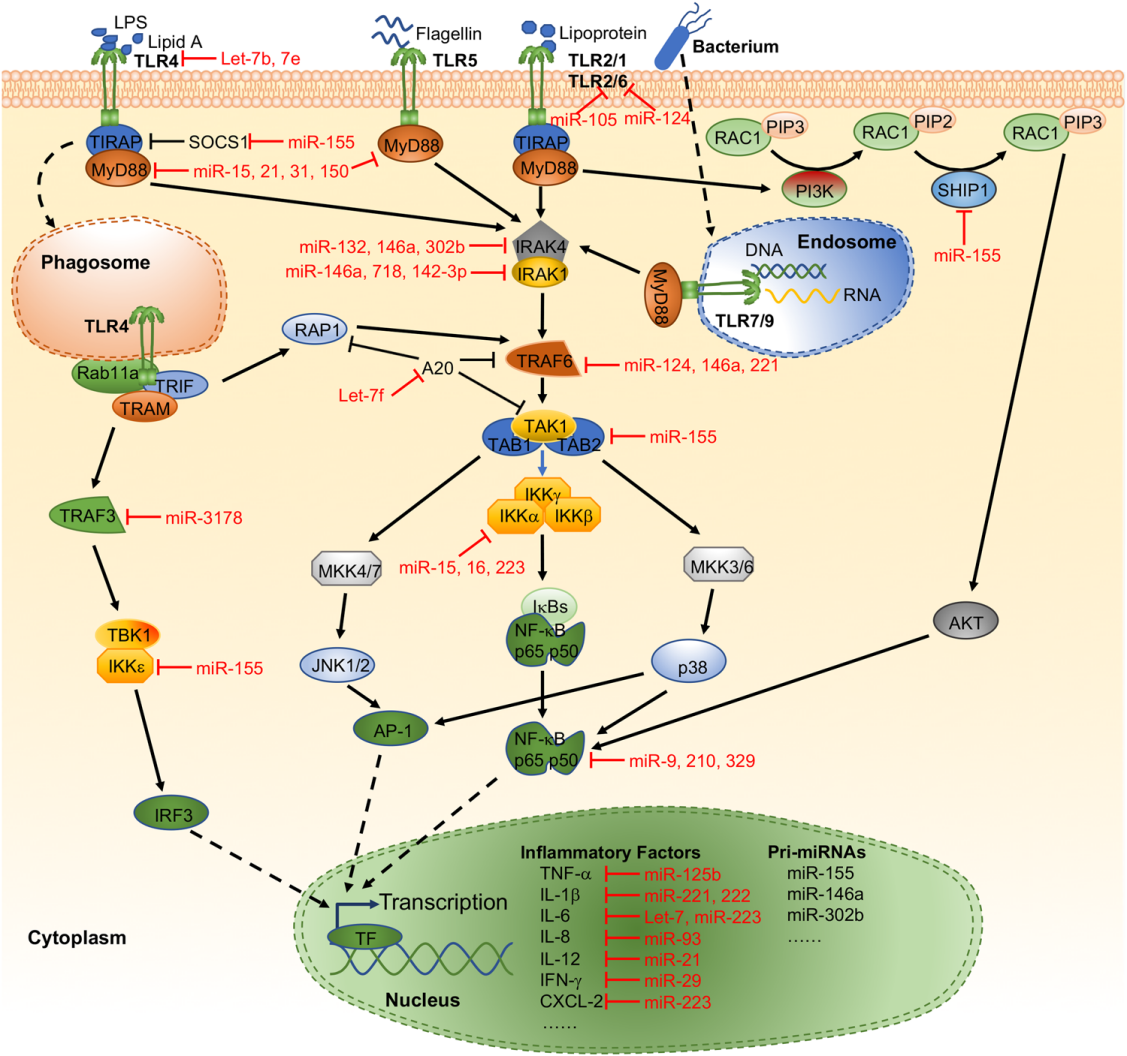
Representative miRNAs in the regulation of TLR signaling. TLRs recognize different bacterial components and induce NF-κB signaling or activate other transcription factors through adapter molecules and downstream signaling molecules. Various inflammatory factors are transcribed that are initiated by different transcription factors. The transcription of miRNAs is most commonly mediated by RNA polymerase II, under the control of transcription factors, and transcripts are then processed by two nucleases, Drosha and Dicer. Then, the mature miRNAs will be incorporated into the RISC and guide the RISC to their target mRNA(s) in cytoplasm. Both early- and late-phase-activated TLRs induce different types and expression levels of inflammatory factors and miRNAs. TF, transcription factor

TLRs were the first identified and the most well-investigated PRRs. TLRs sit at the center of innate immunity against almost any pathogens through their PAMP recognition^3^. After recognizing the PAMPs of pathogens, TLRs can transduce downstream signaling through either MyD88 or TRIF. These TLR-mediated responses may induce secretion of inflammatory cytokines and miRNA expression (Fig. 1). TLR-4 signaling is necessary for enhancing miR-32-5p following Mtb infection. miR-32-5p significantly extended the survival of intracellular mycobacteria^104^. miR-124 was upregulated in the peripheral leukocytes of patients with pulmonary tuberculosis in both *M. bovis-* and BCG-infected macrophages in vitro and *in vivo*. Mechanistically, miR-124 can regulate TLR signaling pathways in macrophages in response to BCG infection^105^. Many subsets of TLRs and related signals, including TLR6, MyD88, TRAF6, and TNF-α, can be directly impacted by miR-124^105,106^. miR-3178 mitigated inflammatory response and gastric carcinogenesis that are facilitated by a *H. pylori* new toxin, Tip-α, via targeting TRAF3^107^. miR-223 may down-modulate NF-κB activation by inhibiting p65 phosphorylation and nuclear translocation^108^. miR-329 plays a major part in promoting trophoblast apoptosis induced by *S. pneumoniae* peptidoglycan (PDG) and inhibiting IL6 mRNA expression involving the NF-κB subunit p65^109^. miR-210 targets another subunit of NF-κΒ, p50, to inhibit LPS-induced expression of proinflammatory cytokines^110^. TLR4-activated NF-κB rapidly increases the expression of miR-9 to provide feedback to NF-κB-dependent responses by fine tuning the expression of the NF-κB subunit p50^111^. IKKα mRNA was targeted by miR-15a, miR-16, and miR-223, which then substantially decreased NF-κB p52 production^112^. miR-146a could also directly target TRAF6 after transcription and ameliorate the activation of NF-κB and p38 MAPK circuits during BCG challenge. miR-146a increase may block inducible nitric oxide (NO) synthase (iNOS) expression and NO generation, thereby enhancing mycobacterial survival in macrophages^78^. In *E. coli*, *Mycobacterium* and *Helicobacter* infection models, miR-146a downregulated the expression of the target genes IRAK1 and TRAF6, thus downregulating LPS levels and bacteria-induced production of cytokines and chemokines, endocytosis and lysosome trafficking^30,31,113–115^. Furthermore, enforced expression of miR-146a generates a tolerance to lipoprotein-, and leading to a significantly decreased expression of in IRAK-1 and phosphorylated IκBα in *S. typhimurium*-infected or control THP-1 cells ^116^.

The involvement of miR-155 in TLR/NF-κB signaling has also been reported. miR-155 downregulated IKK-ε, and increased miR-155 levels downregulated the production of IL-8 and GRO-α in gastric epithelial cells after *H. pylori* infection^24^. SHIP1 is found to be a main target of miR-155, and knocking down endogenous SHIP1 led to AKT activation in response to LPS^117^. In addition, miR-142-3p may have a role in inhibiting proinflammatory mediators NF-κB p50, TNF-α, and IL6 in BCG-challenged macrophages, possibly via targeting IRAK-1^118^. Finally, the TLR adapter MyD88 may also serve as a target of miRNAs. miR-150 targets MyD88, thus leading to suppression of TLR responses in macrophages^66^. miR-214 inhibited the production of inflammatory cytokines by targeting MyD88 to avoid excessive inflammation in *Vibrio harveyi*-infected and LPS-treated fish ^100^.

Several miRNAs can directly target TLRs to modulate immunologic processes. Importantly, Let-7b is partially complementary to the TLR4 mRNA 3′-UTR and therefore influence TLR4 levels at the posttranscriptional levels in gastric epithelium. Overexpression of let-7b reduced TLR4 and subsequently mitigated *H. pylori-*induced activation of NF-κB, MyD88, NF-κB1/p50, and RelA/p65^15^. In macrophages, AKT1 regulates LPS-induced let-7e expression, and let-7e regulates endotoxin sensitivity and tolerance by interacting with the 3’-UTR of TLR4^119^. miR-124 could also play a negative regulatory role for inflammatory responses in macrophages upon mycobacterial infection by directly targeting TLR6^105,106^. In a *Porphyromonas gingivalis* infection model, miR-105 had complementarity for TLR-2 mRNA and inhibited TLR-2 protein translation in human gingival keratinocytes^120^. TLR2 could also be targeted by miR-23a-5p to modulate survival of mycobacteria and activation of autophagy pathways ^121^.

In addition to the well-investigated miRNAs targeting positive signaling cascade molecules, miRNAs could also target negative regulators during bacterial infection. For example, Mtb-triggered let-7f could target A20, which can inhibit NF-κB ^81^.

### Autophagy

**Fig. 2:**
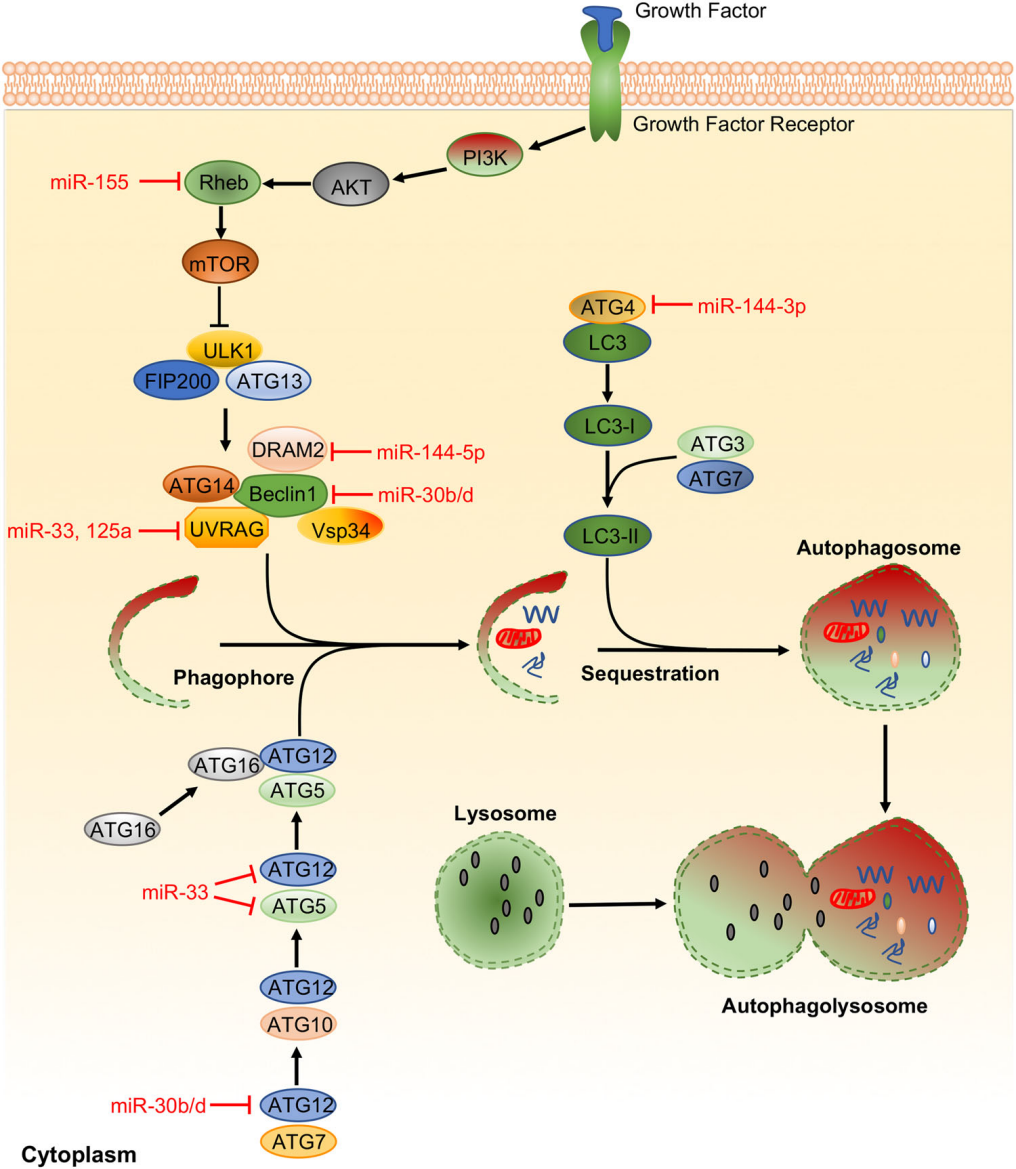
Representative miRNAs in the regulation of autophagy. Autophagy is an important immune response used to eliminate bacterial pathogens. In turn, bacterial pathogens have also developed the ability to subvert host autophagy by interfering with autophagy signaling. Deregulation of miRNAs can occur as the result of interplay between bacterial factors and autophagy components. miRNAs that target autophagy-related proteins function as a specialized immunologic effector and effectively regulate host innate immune responses for the elimination of pathogenic bacteria

Autophagy is a vital measure of eukaryotic cells that maintains cellular homeostasis to recycle nutrients and degrade damaged or aged cytoplasmic constituents, which also impacts the survival of bacterial pathogens^103^. It has become increasingly recognized that abnormal autophagy may contribute to defense against bacterial infection (Fig. 2). BCL2-interacting coiled-coil protein (BECN1) and autophagy-related protein 12 (ATG12) are two important proteins that guide other autophagy proteins to the pre-autophagosomal membrane and subsequently facilitating phagophore elongation and autophagosomes maturation^17^. Two groups have reported that *H. pylori* infection increases miR-30b/d and that compromised autophagy by miR-30b/d promotes bacterial replication by targeting BECN1 and ATG12^17,122^. DNA damage-regulated autophagy modulator 2 (DRAM2) is shown to interact with BECN1 as a coordinator of autophagy activation. miR-144-5p inhibited antimicrobial responses against Mtb in human monocytes and macrophages by targeting DRAM2 ^123^.

These data demonstrated that mycobacterial challenge may augment miR-155 levels, and miR-155 increase induces autophagy through targeting Ras homolog enriched in brain (Rheb), a negative autophagy modulator. miR-155 activates autophagy and facilitating phagosome maturation after mycobacterial internalization into macrophages, thus killing intracellular mycobacteria^67^. However, another group reported no difference in formation of autophagosomes in WT control and miR-155^−/−^ macrophages after virulent Mtb infection by assessing LC3I to LC3II conversion. Their results suggest that inhibiting SHIP1 via miR-155 improves viability in macrophages and reduces bacterial burdens^71^. In contrast, miR-125a could target UV radiation resistance-associated gene (UVRAG) to inhibit autophagy activation and antimicrobial responses to Mtb^75^. Mtb utilized miR-33 to downregulate autophagy and reshape lipid metabolism in hosts to enhance intracellular survival and persistence by influencing critical autophagy components, such as ATG5, ATG12, and UVRAG^124^. Mtb infection could also lead to inhibition of miR-17 and concomitant increase of its targets, myeloid cell leukemia sequence 1 (Mcl-1) and signal transducer and activator of transcription 3 (STAT3), a transcriptional activator of Mcl-1. miR-17 overexpression reduced phosphorylation of protein kinase C delta (PKCδ) and attenuated autophagy during Mtb infection^125^. Furthermore, BCG-challenged macrophages exhibited higher levels of miR-144-3p, which targets ATG4a and inhibits antimicrobial defense^126^. Collectively, these findings suggest that dysregulated miRNAs may be the result of a complex interplay between bacterial factors and autophagy components (Fig. 2).

### Apoptosis

**Fig. 3:**
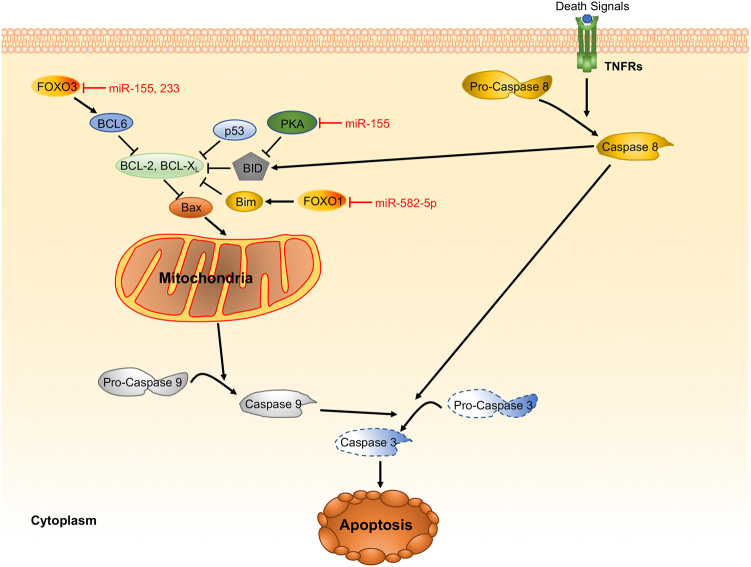
Representative miRNAs in the regulation of apoptosis. Apoptosis has been observed as a response to infection by a wide range of bacterial pathogens. Bacteria can activate several proapoptotic proteins and miRNAs to induce apoptosis. Deregulated miRNAs in infection by bacterial pathogens are involved in networks that control innate immunity and apoptosis pathways of their host cells

Apoptosis is considered as programmed cell death that may play a part in host defense against pathogens. Apoptosis is typically identified with cell shrinkage, DNA fragmentation, mitochondrial permeability, membrane blebbing, and particularly activated caspases (caspase 3)^127^. Intracellular pathogens may modulate host apoptosis signaling to facilitate their proliferation and evade host defenses. Indeed, numerous miRNAs have been reported to regulate apoptosis-related genes during bacterial infection (Fig. 3). Three highly regulated genes (Lpin1, Pmaip1, and Tspan14) associated with apoptosis/DNA damage have been validated as the targets of miR-155 in *H. pylori*-infected BMMs. miR-155^−/−^ BMMs succumbed to apoptosis upon cisplatin treatment versus wild-type counterparts. Furthermore, *H. pylori*-infected miR-155^−/−^ BMMs showed poly (ADP ribose) polymerase (PARP)/procaspase-3 cleavage levels similar to controls^21^. BCG challenge-augmented miR-155 was dependent on TLR2-associated signals. miR-155 also impacts PKA signaling axis by targeting PKI-α, which is a negative modulator of PKA. Notably, miR-155-driven PKA circuit enhanced apoptotic effector activities, thus leading to apoptosis in BCG-infected macrophages^128^. Huang et al. reported that miR-155 inhibited apoptosis of monocytes by targeting Forkhead Box O3 (FOXO3) using an Mtb infection model. Interestingly, miR-233 has similar functions with miR-155 by targeting the same gene^129,130^.

miR-582-5p highly expressed in monocytes was found to be increased in patients with active TB. THP-1 cells transfected with miR-582-5p mimics were much less apoptotic than the cells transfected with a negative control, suggesting miR-582-5p being an inhibitor of apoptosis, which may be through inhibition of FOXO1 expression. Via this mechanism, miR-582-5p should impact immune responses to Mtb infection^131^. MPT64, a secreted protein by Mtb also impeded apoptosis of RAW264.7 macrophages via a NF-κB/miR-21/BCL-2 mechanism. NF-κB was assumed to be the chief transcription factor for miR-21-mediated gene regulation. miR-21 then facilitates BCL-2 expression to block apoptosis^132^. These results suggest that miRNAs may participate in control of bacteria-mediated apoptosis.

### Crosstalk between miRNAs and signaling pathways

As mentioned above, one miRNA may target many mRNAs, and a single mRNA may also be regulated by several or many miRNAs. The target mRNAs of one miRNA usually involve different signaling pathways, and one miRNA can be induced by different PAMPs. It has been speculated that there is a common signaling crosstalk mechanism for mounting protective immune responses against infection. For instance, it has been reported that TLR signaling links the autophagy pathway to phagocytosis and apoptosis in macrophages^133,134^. It appears that TLR2 and MyD88 are critical to the biogenesis of miR-125a during Mtb infection. miR-125a inhibited the induction of autophagy in macrophages by suppressing UVRAG protein expression, which is essential for the coordination of autophagic maturation and endocytic trafficking^75^. Wu et al. showed that SP110 nuclear body protein downregulated miR-125a in macrophages, but upregulated Bcl2-modifying factor (Bmf), an apoptosis-inducing protein. miR-125a interacts with the Bmf 3′-UTR to inhibit Bmf expression, which suppressed Mtb-induced macrophage apoptosis^82^. As we can see from the above description, miRNA roles in bacterial infection are mainly involved in host immune responses against pathogens, which are different from those in several other diseases, such as cancers. Indeed, the same miRNA may play a different role in different models through the same target(s). miR-146a could negatively regulate NF-κB activation by inhibiting the expression of IRAK1 and TRAF6, significantly inhibiting breast tumor growth^135^. It can also modulate innate immune responses via targeting IRAK1 and TRAF6 in bacterial infection models^115^. This functional diversity of miR-146a may be caused by crosstalk between different signaling pathways. Therefore, better understanding of the crosstalk mechanisms underlying the deregulation of miRNAs during bacterial infection is crucial for improving our understanding of immune signaling.

## MiRNAs as biomarkers for bacterial infection

Diagnosis of infection is important for the control of the spread of bacterial invasion and for effective treatment of infection^136^. It is demonstrated that miRNAs are stable in patients’ serum. miRNAs are hard to degrade by RNases, and hence can be used as invaluable biomarkers to detect bacterial infection at earlier stages. Studies showed that 97 miRNAs expressed in a unique manner in pulmonary TB patient sera versus healthy individuals (90 increase while 7 decrease). Among these changed products, miR-361-5p, miR-889 and miR-576-3p stood out to differentiate TB patients from normal subjects or different pathogenic infections^136^. In another study, a larger size of subjects (326 serum samples) were enrolled to search for biomarkers for TB infection in the lung. The authors identified six different miRNA products (miR-101, miR-22, miR-29c, miR-320b miR-378, and miR-483-5p) in the serum as specific biomarkers to diagnose TB lung infection versus normal subjects. A reasonable sensitivity (~95.0%) and specificity (~91.8%) was achieved by combining the 6 miRNAs versus using single miRNA ^137^.

Nevertheless, studying miR-29a expression in human TB cases is still controversial. Fu et al. employed specific miRCURY LNA microarrays to compare the levels of circulating miRNAs between patients with active pulmonary TB and their healthy counterparts. The analysis of the ROC curve showed that upregulated miR-29a may distinguish TB patients from healthy controls^138^. In contrast, another group reported decreased expression of miR-26a, miR-29a, and miR-142-3p in whole blood in children with tuberculosis compared to healthy children with latent Mtb infection (LTBI)^139^. However, Awuah et al. concluded that although median miR-29a expression was slightly higher in TB patients, there was no significant difference compared with LTBI patients^140^. These plausible results might be due to choices of patients and starting material for miRNA quantification.

The miRNA expression profile may have several differences between children and adults. The diagnostic value of miRNA-31 in peripheral blood mononuclear cells (PBMCs) of sixty-five children with pulmonary tuberculosis has been reported. Studies have shown that miRNA-31 in pediatric TB patients exhibited less expression compared to that in normal controls. Importantly, serum miRNA-31 levels correlated inversely with production of IL6, TNF-α, NF-κB, and IFN-γ ^141^.

Multidrug-resistant (MDR) Mtb could result in extended, complicated disease, leading to increased treatment costs or treatment failure. A comparative miRNA analysis was conducted and indicated 142 miRNAs expressed in a different manner between an MDR Mtb strain and a sensitive Mtb strain, (48 increased while 94 decreased). Importantly, only six miRNAs were similarly expressed between the MDR and sensitive Mtb strains, whereas 108 miRNAs were only observed in the MDR Mtb strain^142^. Profiling miRNAs was carried out in plasma samples from cavitary pulmonary tuberculosis (CP-TB) patients, non-cavitary pulmonary tuberculosis (NCP-TB) patients and normal individuals, which revealed candidate biomarkers (miR-769-5p, miR-320a and miR-22-3p) for diagnosis of TB. Additionally, miR-320a may be useful for diagnosing drug-resistant TB^143^. miR-155 levels were lower in patients with MDR TB than healthy subjects. Also, this miRNA was increased in treated patients versus naive patients. miR-16 levels were the lowest in serum of MDR TB patients compared to TB-naive, TB-treated and healthy control groups^144^. Thus, analyzing miRNA expression patterns in MDR and drug-sensitive Mtb may reveal novel mechanisms of drug resistance in TB research.

Several other serum miRNAs are also differentially regulated upon bacterial infection as biomarkers. Serum miR-133a was the highest expressed in mice of a cecal pole ligation and puncture sepsis model, and establishing the strong correlations between miR-133a and disease severity, classical markers of inflammation and bacterial infection, and organ failure of patients with sepsis^145^. miR-155 and miR-197 may possess superior values in distinguishing patients with pneumonia and TB from controls^146^. miR-144 levels in sputum and serum were shown to be increased in TB patients, but markedly decreased following anti-Mtb treatment^147^. miR-155 and miR-155* preferentially expressed in PBMCs of tuberculosis patients showing Mtb-specific antigen, indicating their diagnostic potential for specific Mtb antigens^148^. Moreover, miR-155 was increased in both early (week 2) and late (week 11) *M. bovis-*infected cattle, whereas upregulation was only detected in late stages of BCG-vaccinated cattle. This suggested that miR-155 may be used as a prognostic marker for distinguishing vaccinated from controls^149^. miR-183 was increased in serum samples from TB patients versus healthy subjects. Interestingly, miR-183 expression was positively correlated with macrophage function, as shown by their augmented phagocytosis and enzymatic activity in the group with high serum miR-183 ^150^.

Further studies have also profiled miRNA expression in other clinical samples. Let-7c expression decreased in samples ranging from non-atrophic gastritis to atrophic-metaplastic gastritis, intra-epithelial neoplasia, and invasive GC. It increased again significantly following *H. pylori* eradication^14^. After the identification of miRNAs and their target genes in normal gastroduodenal biopsy, *H. pylori*-infected gastroduodenal biopsy and *H. pylori*-infected gastroduodenal ulcer biopsy samples, Cheng et al. found that increase in miR-155 and miR-146b could decrease *H. pylori*-induced IL6 expression in gastroduodenal ulcer. This relationship between miR-155 and miR-146b and IL6 might reduce the clearance of *H. pylori* and contribute to ulcer development and maintenance^151^. Exosomes, as a rich source of miRNAs, can protect miRNAs from degradation, and have the potential to be a very promising biomarker. Sun et al. detected miRNA levels in bovine milk exosomes derived from lactating Holstein cows infected by *S. aureus*. They reported that bta-miR-142-5p and bta-miR-223 expressed differently as exosomal miRNAs, and may be potential biomarkers for the early diagnosis of bacterial infection, particularly for mammary glands ^152^.

Single-nucleotide polymorphisms (SNPs) in the processing sites of miRNAs may affect miRNA expression and function, which are involved in the pathogenesis of infectious diseases^153,154^. However, different ethnic groups may have distinct attributes for this type of genetic epidemiological research. For instance, the miR-499 rs3746444 T > C rather than miR-146a rs2910164 C > G likely led to increased infection incidence in the lung in Uygur Chinese. Kazak Chinese exhibited a marked different SNP frequency compared to Uygur (miR-146a C > G, but not miR-499 T > C). These same SNPs may also be related to Mtb in Tibetan people. Despite discrepant reports, the analysis of mRNA SNPs may be not a good measure for evaluating Mtb sensitivity in Southern Han Chinese ^155,156^.

miRNAs may serve as a valuable diagnostic marker that may be useful only for specific bacterial infections. Accumulating evidence suggests that almost all the miRNA biomarkers evaluated to date have been for the diagnosis of pulmonary tuberculosis and *H. pylori*-associated gastritis, which may reflect differences in the field difference due to intense interest in these bacteria. Pulmonary tuberculosis, *H. pylori*-associated gastritis and other specific bacterial diseases with clear induction factors would be more suitable for early diagnosis via miRNA expression profiling. However, the accuracy of detecting miRNA levels among different samples remains quite challenging. A study of whole-blood miRNA features for the effective early diagnosis of pulmonary tuberculosis revealed that evaluating certain specialized miRNAs in combination could be accomplished with reasonable sensitivities and specificities ^157^.

## MiRNAs as therapeutic targets for bacterial infection

The revelation that miRNAs function as an important regulator in bacterial infection suggests their great application potential as novel therapeutic targets. Indeed, many miRNAs have been developed and investigated in clinical trials for several types of diseases. The first miRNA-based drugs, MRX34, entered a phase 1 trial in patients with primary liver cancer or metastatic cancer in 2013^158^. Miravirsen, a 15-nucleotide locked nucleic acid-modified antisense oligonucleotide that can sequester and thus inhibit miR-122, could induce significant virologic responses after subcutaneous injections in patients with chronic HCV infection^159^. Though there are no bacterial infection-related miRNA-based drugs evaluated in clinical research to date, miRNAs still represent a promising approach for future therapies or as immune modulators against invading pathogens. Several miRNAs with excellent immune regulation efficiency, such as miR-33^124^, miR-155^44^, miR-29^65^, miR-146a^115^, and miR-302b^52^, may have great developmental potential for further clinical studies.

The application of miRNA-based therapeutics still faces many hurdles before it can be translated into clinical practice for bacterial infections. The spatio-temporal expression of miRNAs and their targets at different stages during infection must first be precisely identified. If the miRNAs that can target numerous genes can be specifically designed, this could represent a significant advantage of miRNA-targeted approaches. As the multicenter phase I clinical study of MRX34 was terminated due to five immune-related serious adverse events last year, the efficacy and safety of miRNA-based drugs need to be carefully assessed^160^. The tissue/target specificity delivery and stability of miRNA-based drugs is another current limiting factor for satisfactory therapeutic effects. In addition to well-investigated virus and non-virus delivery systems, recent reports indicate that exosomes may be an emerging high-efficiency delivery approach ^161,162^.

## Conclusion and perspectives

miRNA research has provided a unique angle for studying the mechanisms underlying how the innate immune system senses and responds to microbial pathogens. Over the past decade, the role of miRNAs in bacterial pathogen infection has greatly enhanced our understanding of cellular physiology and immunology. In particular, miR-155 and miR-146 are the two most well-understood miRNAs, with well-characterized roles in immunity and inflammation regulation during bacterial infection.

However, due to the complexity of bacterial infections, one miRNA may regulate several different targets in different stages during bacterial infection. The precise mechanism underling the regulatory function of miRNAs must be explored. Future studies are needed to further clarify how miRNAs with diverse targets may affect overall host responses during infection. In certain instances, the continuous development of pathological processes is accompanied by changes in the miRNA expression profile. It is necessary to distinguish that are the most important regulators and which intervention strategy is the most effective.

Bacterial pathogens have developed a variety of virulence factors to facilitate bacterial colonization in their hosts, invade deeper tissues and evade host defenses^163,164^. Currently, researchers have only identified bacterial infections that can cause changes in the expression of a variety of miRNA. Many miRNAs could be induced during infection with different types of bacteria. It has been noted that several aberrant miRNAs during bacterial infection are also dysregulated in other diseases. For example, levels of miR-155 are altered upon infection with *P. aeruginosa*, *Mtb* and *H. pylori*, and miR-155 plays a role in host immune responses^23,54,71^. Many reports have shown that miR-155 is involved in several other diseases, such as rheumatoid arthritis^165^, breast cancer^166^, atherosclerosis^167^, Crohn’s disease^168^, and others. The specificity of miRNA expression induced by certain bacteria or their virulence factors requires more investigation. This would be of the utmost importance for the application of unique miRNAs for diagnosis and treatment.

Furthermore, mutations within miRNAs may also alter their target selection, thereby preventing them from inhibiting tuberculosis-related genes, thus increasing host susceptibility to disease. Amila et al. investigated the genetic association of pulmonary tuberculosis with six human miRNA genes that have been predicted to interact with tuberculosis genes. However, this study did not show differences in the sequences compared with healthy individuals without antecedents of tuberculosis^169^. Despite the negative results in this study, larger samples are needed for clarification in future studies, especially in bacterial pathogen-susceptible or resistant populations. Even in the absence of clinical symptoms, several host-adapted bacterial pathogens (e.g., Mtb, *S. typhimurium* and *H. pylori*) are capable of maintaining infections in mammalian hosts even in the presence of inflammation, specific antimicrobial mechanisms and a robust adaptive immune response, which may be due to persistent infections^170,171^. However, the role of miRNAs in the fundamental genetics of bacterial persistence in the presence of immuno-surveillance has only recently begun to be clarified.

The relationship between miRNAs and bacterial pathogens and the underlying mechanisms urgently require broader investigation. Hence, the elucidation of the function of miRNAs on host–pathogen interactions may lead to the discovery of novel and effective preventive measures and the development of rational therapeutic strategies.
